# Circulating angiogenic T cells as potential immune-vascular biomarkers associated with disease activity in axial spondyloarthritis

**DOI:** 10.3389/fimmu.2026.1886629

**Published:** 2026-09-28

**Authors:** Wei Zhou, Shipei Zhang, Linting Yin, Meizi Liu, Shenghui Liu, Lin Hua, Guohui Xue

**Affiliations:** 1 Department of Clinical Laboratory, Jiujiang First People’s Hospital, Jiujiang, Jiangxi, China; 2 Jiujiang City Key Laboratory of Cell Therapy, Jiujiang First People’s Hospital, Jiujiang, Jiangxi, China; 3 Department of Rheumatology and Immunology, Jiujiang City Key Laboratory of Cell Therapy, Jiujiang First People’s Hospital, Jiujiang, Jiangxi, China

**Keywords:** angiogenic T cells, axial spondyloarthritis, disease severity, immune-vascular biomarker, immunophenotyping

## Abstract

**Objective:**

Axial spondyloarthritis (axSpA) is a chronic inflammatory autoimmune disease associated with persistent systemic inflammation and endothelial dysfunction. Angiogenic T cells (Tang cells), defined as CD3^+^CD31^+^CD184^+^ T cells, are specialized immune cells involved in vascular repair and endothelial homeostasis, but their clinical relevance in axSpA remains unclear. This study aimed to characterize circulating Tang cells in axSpA and evaluate their potential as immune-vascular biomarkers of disease activity.

**Methods:**

Peripheral blood samples were obtained from patients with axSpA stratified by disease activity and from healthy controls. Circulating Tang cells and their phenotypic subsets were quantified using flow cytometry. Clinical characteristics, disease activity indices (ASDAS and BASDAI), inflammatory markers, and immunometabolic parameters were collected. Correlation analyses, multivariable logistic regression, and receiver operating characteristic (ROC) curve analyses were performed to assess the clinical significance of Tang cells in relation to axSpA disease activity.

**Results:**

Tang cell frequency and absolute count were significantly decreased in active axSpA (median 16.40% [11.97%-25.65%]; 195.82 [135.07-322.53] cells/µL) compared with stable axSpA (32.11% [24.62%-42.33%]; 442.89 [306.48-633.02]) and healthy controls (32.23% [26.57%-43.87%]; 437.95 [293.73-507.95]) (all *P* < 0.001). Tang cell frequency showed a positive correlation with CD14^+^HLA-DR^+^ monocyte proportions (Spearman r = 0.537, *P* < 0.001) and an inverse correlation with BASDAI scores (Spearman r = −0.548, *P* < 0.001). Multivariable regression analysis revealed that circulating Tang cell frequency remained independently and inversely associated with active disease status (OR = 0.87, 95% CI: 0.79 - 0.95, *P* = 0.002) after adjustment for traditional inflammatory markers including erythrocyte sedimentation rate (ESR) and C-reactive protein (CRP). In addition, circulating Tang cells showed satisfactory discriminatory ability to distinguish active axSpA from stable axSpA (area under the curve [AUC] = 0.831).

**Conclusions:**

Circulating angiogenic T cells are inversely associated with disease activity in axSpA and represent a novel immune-vascular biomarker that is not redundant with traditional inflammatory parameters. These findings provide a clinical immunophenotypic framework highlighting altered vascular repair-immune interactions in axSpA and support further functional studies to clarify the functional role of Tang cells in disease pathogenesis.

## Introduction

Axial spondyloarthritis (axSpA) is a chronic inflammatory autoimmune disease that primarily affects the sacroiliac joints and axial skeleton, with typical onset in young adults. Its pathogenesis involves a complex interplay of genetic susceptibility, immune dysregulation, and aberrant bone remodeling (1). Clinically, axSpA manifests as chronic inflammatory back pain, morning stiffness, and progressive structural damage, including pathological new bone formation and ligament ossification, which can culminate in the characteristic “bamboo spine” appearance on imaging (2). These irreversible changes substantially impair physical function and quality of life and impose a significant healthcare burden (3). Despite advances in anti-inflammatory therapies, reliable immunological markers that reflect disease activity and underlying immune alterations in axSpA remain limited.

Beyond musculoskeletal involvement, axSpA is increasingly recognized as a systemic inflammatory disease with extra-articular manifestations such as uveitis and inflammatory bowel disease (4, 5). Persistent systemic inflammation can adversely affect the vascular endothelium, leading to endothelial dysfunction, an early event in the development of cardiovascular pathology (6–8). In axSpA, proinflammatory cytokines, particularly tumor necrosis factor-α (TNF-α) and interleukin-17 (IL-17), directly impair endothelial cell function by increasing vascular permeability, enhancing adhesion molecule expression, and promoting a pro-inflammatory endothelial phenotype (9, 10). These findings underscore the close interplay between immune activation and vascular homeostasis in axSpA.

Immune cell dysregulation is a central feature of the inflammatory milieu in axSpA (11). Distinct T-cell subsets, including regulatory T cells, Th17 cells, and other pathogenic or unconventional effector cells such as γδ T cells and mucosal-associated invariant T cells, have been implicated in disease activity and immune imbalance (12–14). In addition to these well-characterized populations, angiogenic T cells (Tang cells) represent a specialized T-cell subset involved in endothelial repair and vascular homeostasis (15). Phenotypically defined by the co-expression of CD3, CD31 (PECAM-1), and CD184 (CXCR4), Tang cells support endothelial regeneration through the secretion of pro-angiogenic mediators and direct interactions with endothelial cells (16). Altered circulating Tang cell frequencies have been reported in several autoimmune and inflammatory diseases, including rheumatoid arthritis, systemic lupus erythematosus, and systemic sclerosis, where they are associated with inflammatory burden and disease activity (17–19). However, their relevance in the immunopathology of axSpA has not been systematically explored.

Given the recognized links between systemic inflammation, immune dysregulation, and endothelial impairment in axSpA, characterization of circulating Tang cells may provide insight into immune-vascular alterations accompanying disease activity. Therefore, the present study aimed to quantitatively characterize circulating Tang cells in patients with axSpA using multiparameter flow cytometry and to examine their associations with clinical disease activity indices and inflammatory markers. By focusing on immunophenotypic changes related to disease activity, this study evaluates the potential of Tang cells as clinical immunological biomarkers and establishes a framework for future investigations into their functional roles in axSpA.

## Materials and methods

### Study population

This study consecutively enrolled hospitalized patients diagnosed with axSpA from the Departments of Rheumatology and Immunology and Orthopedics at Jiujiang First People’s Hospital. Patients with established axSpA receiving long-term maintenance therapy were enrolled during hospitalization for acute disease flares, primarily presenting with severe pain, marked functional impairment, or disease-related complications. Healthy individuals undergoing routine physical examinations at the hospital’s Health Management Center during the same period were recruited as controls. A total of 54 patients with axial spondyloarthritis (axSpA) were included, all of whom fulfilled the 2009 ASAS classification criteria for axial spondyloarthritis (20). Patients with acute infections, other active systemic autoimmune diseases, malignancy, or severe organ dysfunction were excluded from the study. Patients were stratified according to disease activity based on the Bath Ankylosing Spondylitis Disease Activity Index (BASDAI): a stable axSpA group (BASDAI < 4, n = 22) and an active axSpA group (BASDAI ≥ 4, n = 32). The most recent Axial Spondyloarthritis Disease Activity Score using C-reactive protein (ASDAS) was also obtained. Healthy controls (HCs, n = 30) were enrolled with no history of autoimmune or inflammatory diseases and no abnormal findings on physical examination. Demographic characteristics, comorbidities, and clinical data were systematically retrieved from the hospital’s electronic medical record system. This study was approved by the Ethics Committee of Jiujiang First People’s Hospital (registration number: JJSDYRMYY-YXLL-2024-246), and all procedures were conducted in accordance with the Helsinki Declaration.

### Sample collection and processing

Peripheral venous blood samples were collected from all participants within 48 hours of hospital admission. Blood samples were drawn into tubes containing ethylenediaminetetraacetic acid (EDTA), sodium citrate, or no anticoagulant, as appropriate. EDTA-anticoagulated whole blood samples were transported immediately to the laboratory and processed for flow cytometric analysis within 4 hours of collection. Serum and plasma were separated by centrifugation at 2, 000 × g for 10 minutes at 4 °C and stored −80 °C until further analysis of inflammatory and immunometabolic parameters.

### Laboratory measurements

Routine hematological parameters, including white blood cells, red blood cells, hemoglobin, platelets, neutrophils, lymphocytes, and monocytes, were measured using an automated hematology analyzer (Mindray BC-5380). Biochemical indices, including alanine aminotransferase, aspartate aminotransferase, blood urea nitrogen, creatinine, uric acid, glucose, triglycerides, total cholesterol, high-density lipoprotein, low-density lipoprotein and apolipoprotein E were assessed using an automated biochemical analyzer (Siemens Advia 2400). Serum levels of C-reactive protein, complement C3, and complement C4 were measured by immunoturbidimetric assays using the IMMAGE 800 Immunochemistry System. The erythrocyte sedimentation rate (ESR) was measured using a ZC60 fully automatic ESR and hematocrit analyzer (Beijing Zhongchi Weiye Technology Co., Ltd., Beijing, China). These laboratory parameters were used to characterize the systemic inflammatory and metabolic profiles in the study population.

### Flow cytometry analysis

Flow cytometric analyses were performed on EDTA-anticoagulated whole blood samples using a Beckman DxFLEX flow cytometer within 4 hours of sample collection. For immunophenotyping, two commercial antibody kits were used: CD45-FITC/CD56-PE/CD19-ECD/CD3-PC5 and CD45-FITC/CD4-PE/CD8-ECD/CD3-PC5 (catalogue Nos. 6607013 and 6607073, respectively). These panels enabled the quantification of CD3^+^ T cells, CD3^+^CD4^+^ helper T cells, CD3^+^CD8^+^ cytotoxic T cells, CD3^-^CD19^+^ B cells, and CD3^-^CD56^+^ NK cells. A standardized flow cytometry gating strategy was employed. Briefly, after excluding debris and non-lymphocytes by light scatter (FSC/SSC), CD45^+^ lymphocytes were first identified based on CD45-PC7 expression. From this population, CD3^+^ T lymphocytes were gated based on CD3-ECD expression. Subsequently, CD3^+^CD31^+^ T cells were selected from the CD3^+^ gate using CD31-FITC. Finally, CD3^+^CD31^+^CD184^+^ Tang cells (angiogenic T cells), the effector cells of interest, were specifically identified within the CD3^+^CD31^+^ population using CD184-PE. The frequencies of Tang cells were calculated as a percentage of total CD3^+^ T lymphocytes. Their absolute counts were then derived by applying these frequencies to the absolute count of CD3^+^ T cells, which was determined using fluorescent counting beads. Regulatory T cells (Tregs) were defined as CD3^+^CD4^+^CD25^high^CD127^low^ cells with the antibody panel: CD3-ECD, CD4-FITC, CD25-APC-A700, CD127-PE, and CD45-PC7. As a marker of immune activation, HLA-DR expression on monocytes was assessed using an antibody panel containing CD45-PC7, HLA-DR-PE, and CD14-ECD.

### Statistical analysis

Statistical analyses were performed using SPSS version 24.0 (IBM Corp.), and graphical representations were generated using GraphPad Prism version 8.0.2. Data normality was assessed using the Shapiro-Wilk test. Normally distributed continuous variables are presented as mean ± standard deviation and were compared using independent-samples t tests. Non-normally distributed variables are expressed as median (interquartile range) and were compared using the Mann-Whitney U test. Categorical variables are presented as counts and percentages and were analyzed using the chi-square test. Associations between variables were evaluated using Spearman’s rank correlation analysis. A multivariable logistic regression model was constructed based on predefined clinical relevance criteria to adjust for potential confounders such as age and sex. Receiver operating characteristic (ROC) curve analyses were performed to assess the discriminatory performance of disease activity–related biomarkers, and the area under the curve (AUC) was calculated. A two-tailed *P* value < 0.05 was considered statistically significant.

## Results

### Demographic and clinical characteristics of study participants

The demographic and clinical characteristics of patients with axSpA and HCs are summarized in **Table 1**. A total of 54 patients with axSpA were included, comprising 22 patients with stable disease and 32 patients with active disease. No significant differences were observed among the stable axSpA, active axSpA, and healthy control groups with respect to age or sex distribution (*P* > 0.05). As expected, patients in the active axSpA group exhibited significantly higher ASDAS and BASDAI scores than those in the stable axSpA group (*P* < 0.001). Disease duration and the prevalence of major comorbidities, including hypertension, hyperlipidemia, and cardiovascular comorbidities, were fully balanced between the two axSpA subgroups (*P* > 0.05). As shown in **Table 1**, the baseline proportions of patients receiving b/tsDMARDs (90.91% vs. 75.00%), csDMARDs (9.09% vs. 6.25%), NSAIDs (50.00% vs. 75.00%), glucocorticoids (0.00% vs. 3.13%), and lipid-lowering agents (9.09% vs. 3.13%) did not differ significantly between the stable and active axSpA groups (all *P* > 0.05).

**Table 1:** Demographic, clinical, and medication characteristics of axSpA patients and HCs.

Variables	Stable axSpA (n = 22)	Active axSpA (n = 32)	HCs (n = 30)	*P*1	*P*2	*P*3
Demographic						
Age (years)	32.50 (22.25-51.50)	45.50 (32.50-51.00)	37.00 (31.25-49.25)	0.153	0.568	0.117
Sex (Female/male)	4/18	6/26	6/24	0.869	0.901	0.958
Disease characteristics						
HLA-B27 (+), n (%)	20 (90.90)	29 (90.60)	NA	NA	NA	0.970
Disease duration (years)	4.50 (2.00-12.50)	10.00 (3.25-15.75)	NA	NA	NA	0.248
BASDAI	3.20 (2.80-3.43)	4.90 (4.30-5.28)	NA	NA	NA	<0.001
ASDAS	2.25 (2.10-2.60)	3.10 (2.60-4.50)	NA	NA	NA	<0.001
Comorbidities						
Hypertension, n (%)	2 (9.09)	4 (12.50)	NA	NA	NA	>0.999
Hyperlipidemia, n (%)	1 (4.55)	5 (15.63)	NA	NA	NA	0.405
Cardiovascular Comorbidities, n (%)	1 (4.55)	3 (9.38)	NA	NA	NA	0.891
Baseline medications						
b/tsDMARDs, n (%)	20 (90.91)	24 (75.00)	NA	NA	NA	0.262
csDMARDs, n (%)	2 (9.09)	2 (6.25)	NA	NA	NA	>0.999
NSAIDs, n (%)	11 (50.00)	24 (75.00)	NA	NA	NA	0.059
Glucocorticoids, n (%)	0 (0.00)	1 (3.13)	NA	NA	NA	>0.999
Lipid-lowering agents, n (%)	2 (9.09)	1 (3.13)	NA	NA	NA	0.737

Continuous non-normally distributed data are expressed as median (IQR, Q1-Q3), and categorical variables as n (%). Continuous variables were compared using the Mann–Whitney U test; categorical variables were compared using the Chi-square test or Fisher’s exact test. *P*1, Stable axSpA vs. HCs; *P*2, Active axSpA vs. HCs; *P*3, Active axSpA vs. Stable axSpA. *P* < 0.05 was considered statistically significant. b/tsDMARDs comprised TNF-α inhibitors such as adalimumab and etanercept biosimilars, as well as the IL-17A inhibitor secukinumab. csDMARDs included sulfasalazine, methotrexate, and thalidomide. Other medications included NSAIDs such as celecoxib and etoricoxib, Glucocorticoids such as triamcinolone, and Lipid-lowering agents such as atorvastatin.

ASDAS, Axial Spondyloarthritis Disease Activity Score; axSpA, axial spondyloarthritis; BASDAI, Bath Ankylosing Spondylitis Disease Activity Index; b/tsDMARDs, biologic or targeted synthetic disease-modifying antirheumatic drugs; csDMARDs, conventional synthetic disease-modifying antirheumatic drugs; HCs, healthy controls; HLA-B27, human leukocyte antigen-B27; IQR, interquartile range; NA, not applicable; NSAIDs, nonsteroidal anti-inflammatory drugs.

### Comparison of laboratory parameters among groups

Laboratory parameters were compared among the stable axSpA group, the active axSpA group, and the HC group, with detailed results presented in **Table 2**. With respect to inflammatory and hematological markers, patients with active axSpA demonstrated significantly higher ESR and CRP levels relative to both stable axSpA patients (*P* < 0.001 and *P* = 0.018, respectively) and HCs (*P* = 0.015 and *P* < 0.001, respectively). Monocyte counts were significantly elevated in both axSpA subgroups compared with HCs (*P* < 0.01), whereas hemoglobin (HGB) levels were significantly lower in active axSpA patients than in HCs (*P* < 0.001). Regarding lipid profiles and complement components, active axSpA patients displayed significantly higher APOB, C3, and C4 levels compared with stable axSpA patients (all *P* < 0.05). Conversely, LDL-C levels were significantly lower in both axSpA subgroups than in HCs (*P* < 0.001). Additionally, blood urea nitrogen (BUN) levels were significantly higher in active axSpA patients relative to stable patients (*P* = 0.038).

**Table 2:** Laboratory parameters of study groups based on disease activity.

Variables	Stable axSpA (n = 22)	Active axSpA (n = 32)	HCs (n = 30)	*P*1	*P*2	*P*3
WBC (×10^9^/L)	6.01 (4.68-7.61)	6.12 (5.42-7.52)	5.54 (5.00-6.25)	0.591	0.070	0.570
RBC (×10^12^/L)	4.79 ± 0.60	4.70 ± 0.49	4.85 ± 0.38	0.693	0.204	0.559
HGB (g/L)	143.50 (133.75-150.75)	138.00 (129.00-145.50)	152.50 (143.50-158.75)	0.011	< 0.001	0.433
PLT (×10^9^/L)	223.50 (197.25-249.50)	241.50 (211.75-276.75)	206.50 (179.25-263.00)	0.459	0.056	0.159
NEU (×10^9^/L)	3.53 (2.50-4.57)	3.72 (3.23-4.89)	3.47 (2.74-4.17)	0.846	0.228	0.260
LYM (×10^9^/L)	2.00 (1.55-2.30)	1.55 (1.33-2.25)	1.77 (1.44-2.17)	0.442	0.294	0.118
MON (×10^9^/L)	0.45 (0.35-0.55)	0.42 (0.33-0.57)	0.33 (0.26-0.41)	0.004	0.006	0.826
ALT (U/L)	17.85 (12.62-25.85)	16.65 (11.17-29.30)	19.50 (15.25-22.50)	0.718	0.521	0.758
AST (U/L)	18.55 (16.27-24.03)	18.80 (15.93-22.95)	17.00 (16.00-19.75)	0.121	0.309	0.616
BUN (mmol/L)	4.25 (3.56-5.12)	5.29 (4.16-6.32)	5.42 (4.29-5.95)	0.018	0.886	0.038
CR (μmol/L)	73.10 (61.45-84.43)	69.75 (57.22-75.75)	74.00 (64.50-82.00)	0.650	0.197	0.573
UA (μmol/L)	347.77 ± 87.56	347.75 ± 95.59	359.37 ± 65.84	0.588	0.489	0.819
TG (mmol/L)	0.92 (0.64-1.33)	1.12 (0.81-1.78)	1.08 (0.82-1.49)	0.185	0.994	0.283
TC (mmol/L)	4.05 ± 0.92	4.30 ± 0.86	4.42 ± 0.76	0.120	0.569	0.156
HDL-C (mmol/L)	1.13 (1.05-1.30)	1.16 (0.99-1.28)	1.01 (0.92-1.18)	0.034	0.074	0.585
LDL-C (mmol/L)	2.21 ± 0.60	2.45 ± 0.64	3.11 ± 0.68	< 0.001	< 0.001	0.166
APOB (g/L)	0.65 (0.59-0.92)	0.78 (0.67-0.91)	0.87 (0.78-0.95)	0.021	0.054	0.022
APOE (mg/L)	36.42 ± 9.95	40.20 ± 12.19	39.79 ± 7.91	0.190	0.878	0.255
C3 (mg/dL)	89.30 (76.38-101.42)	99.45 (87.12-118.75)	104.00 (85.25-133.50)	0.023	0.438	0.041
C4 (mg/dL)	19.05 (17.02-24.22)	26.80 (20.58-32.27)	26.15 (19.48-29.85)	0.031	0.602	0.016
ESR (mm/h)	6.00 (3.25-10.25)	14.00 (7.75-32.25)	10.00 (8.00-12.00)	0.734	0.015	0.001
CRP (mg/dL)	0.30 (0.21-1.93)	3.01 (0.40-12.70)	0.41 (0.30-0.68)	0.096	< 0.001	0.018

Continuous non-normally distributed data are expressed as median (IQR, Q1-Q3), and normally distributed data as mean ± SD. Normally distributed continuous variables were compared using Student’s t-test, while non-normally distributed continuous variables were compared using the Mann–Whitney U test. *P*1, Stable axSpA vs. HCs; *P*2, Active axSpA vs. HCs; *P*3, Active axSpA vs. Stable axSpA. *P* < 0.05 was considered statistically significant.

ALT, alanine aminotransferase; APOB, apolipoprotein B; APOE, apolipoprotein E; AST, aspartate aminotransferase; axSpA, axial spondyloarthritis; BUN, blood urea nitrogen; C3, complement component 3; C4, complement component 4; CR, creatinine; CRP, C-reactive protein; ESR, erythrocyte sedimentation rate; HCs, healthy controls; HGB, hemoglobin; HDL-C, high-density lipoprotein cholesterol; IQR, interquartile range; LDL-C, low-density lipoprotein cholesterol; LYM, lymphocytes; MON, monocytes; NEU, neutrophils; PLT, platelets; RBC, red blood cells; SD, standard deviation; TC, total cholesterol; TG, triglycerides; UA, uric acid; WBC, white blood cells.

### Comparison of immune cell subsets

Flow cytometry was performed to analyze the composition of major peripheral blood immune cell populations between axSpA patients across different disease states and healthy controls (**Table 3**). Notably, both the frequency and absolute count of circulating Tang cells were significantly lower in patients with active axSpA than in those with stable disease and healthy controls (all *P* < 0.001; **Table 3**). Conversely, no significant differences were observed between stable axSpA patients and healthy controls (*P* = 0.591 and *P* = 0.493, respectively). Compared with healthy controls, the frequency of regulatory T cells (Tregs) was significantly increased in patients with axSpA, observed in both the active and stable disease states. No significant difference in CD14^+^HLA-DR^+^ cell percentage was observed between the combined axSpA patient groups and healthy controls. However, within the axSpA cohort, the active disease group showed a significantly lower proportion compared to the stable disease group. Conversely, no statistical differences (*P* > 0.05) were found in the conventional immune cell subsets, including the proportions of CD3^+^CD4^+^ T cells, CD3^+^CD8^+^ T cells, CD3^-^CD19^+^ B cells, and CD3^-^CD56^+^ NK cells, suggesting these subsets are relatively stable across active and stable phases and may not directly participate in the dynamic regulation of axSpA activity.

**Table 3:** Immune cell profiles in stable axSpA, active axSpA, and healthy controls.

Variables	Stable axSpA (n = 22)	Active axSpA (n = 32)	HCs (n = 30)	*P*1	*P*2	*P*3
CD3^+^ cells (%)	72.52 (66.35-75.66)	67.45 (62.35-74.95)	70.58 (65.97-74.91)	0.637	0.517	0.369
CD3^+^CD4^+^ cells (%)	35.13 (31.23-40.98)	40.22 (35.99-44.70)	33.64 (30.45-40.29)	0.734	0.037	0.083
CD3^+^CD8^+^ cells (%)	26.45 (21.88-30.36)	23.07 (19.24-28.45)	24.94 (21.32-29.14)	0.760	0.234	0.215
CD3^-^CD19^+^ cells (%)	13.91 (9.52-16.44)	12.48 (10.44-14.25)	11.85 (7.64-12.80)	0.069	0.210	0.679
CD3^-^CD56^+^ cells (%)	12.98 (7.64-17.19)	16.27 (11.16-23.69)	16.29 (11.29-22.14)	0.071	0.916	0.120
Treg (%)	6.33 (4.61-8.06)	5.88 (4.58-6.84)	4.43 (3.88-5.16)	< 0.001	< 0.001	0.458
CD14^+^HLA-DR^+^ cells (%)	93.16 (86.92-96.14)	79.67 (72.12-92.08)	88.59 (78.82-93.00)	0.082	0.083	0.017
Tang cells (%)	32.11 (24.62-42.33)	16.40 (11.97-25.65)	32.23 (26.57-43.87)	0.591	< 0.001	< 0.001
Tang cell counts (cells/µL)	442.89 (306.48-633.02)	195.82 (135.07-322.53)	437.95 (293.73-507.95)	0.493	< 0.001	< 0.001

Continuous data are expressed as median (IQR, Q1–Q3). Continuous variables were compared using the Mann–Whitney U test. *P*1, Stable axSpA vs. HCs; *P*2, Active axSpA vs. HCs; *P*3, Active axSpA vs. Stable axSpA. *P* < 0.05 was considered statistically significant.

axSpA, axial spondyloarthritis; HCs, healthy controls; HLA-DR, human leukocyte antigen-DR; IQR, interquartile range; Treg, regulatory T cells; Tang cells, angiogenic T cells.

### Reduced proportion of circulating Tang cells in patients with active axSpA

The proportions and absolute counts of Tang cells among CD3^+^ lymphocytes were further compared among the stable axSpA, active axSpA, and HC groups. The flow cytometric gating strategy for Tang cell identification is shown in **Figure 1A**. Patients with axSpA showed significant alterations in circulating Tang cell subsets compared with healthy controls, with stratified analyses demonstrating a marked reduction in Tang cells specifically in active axSpA relative to both stable axSpA and healthy controls. (**Figure 1B**).

**Figure 1:**
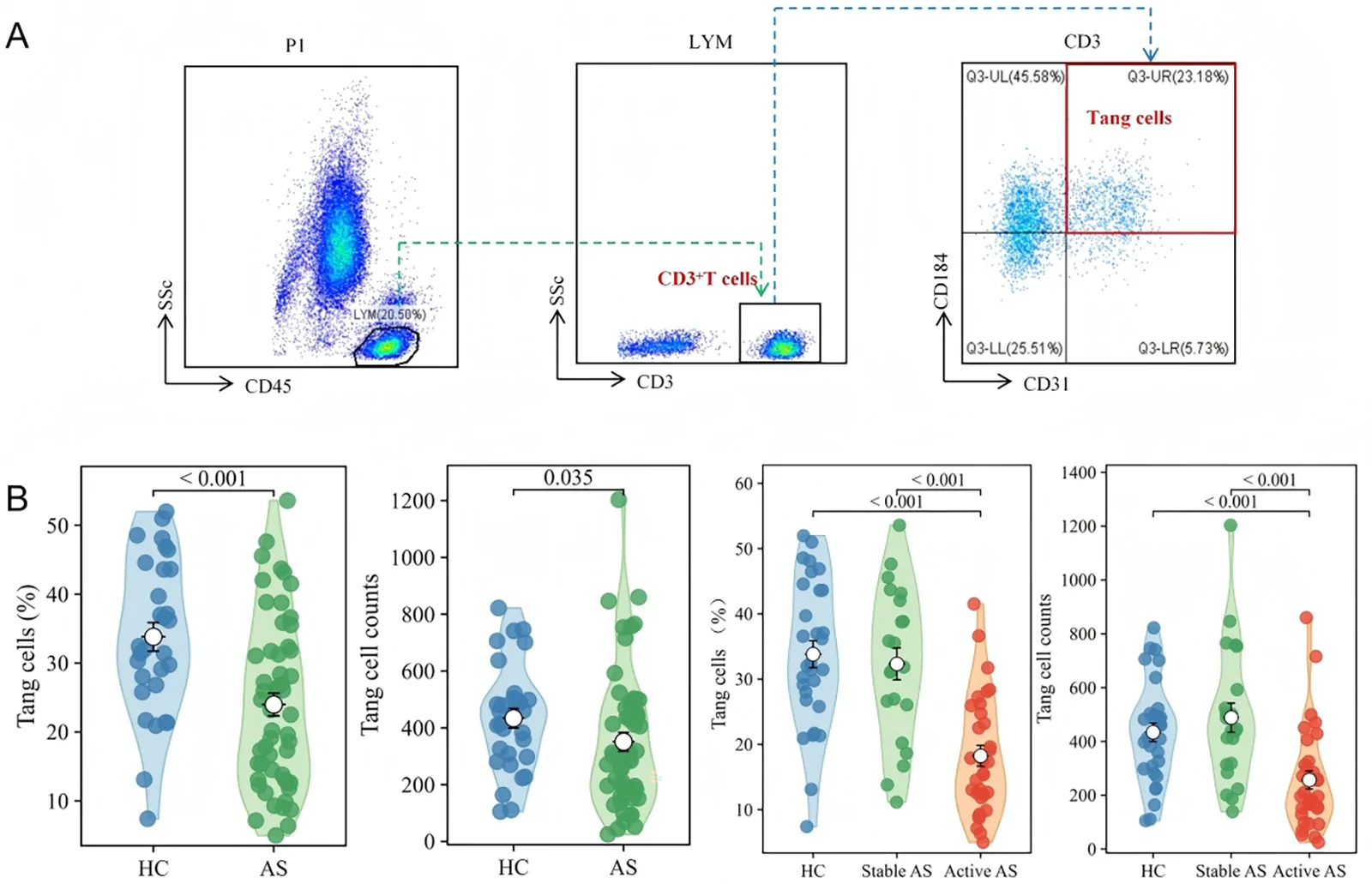
Assessment of Tang cell alterations in patients with axSpA. **(A)** Representative gating strategy for identifying Tang cells. Lymphocytes were first gated based on CD45 expression and side scatter (SSC). T lymphocytes were then selected using CD3 expression versus SSC, and the CD3^+^CD31^+^CD184^+^ Tang cell subset was further identified within the CD3^+^ T-cell population. **(B)** Comparison of Tang cells percentage and counts. Significance was determined using the unpaired Mann-Whitney-Wilcoxon U test. axSpA, axial spondyloarthritis; HC, Healthy Controls; LYM, Lymphocytes; Tang cells, angiogenic T cells.

### Association between Tang cells and disease activity

Spearman correlation analysis demonstrated distinct correlational profiles for Tang cells with clinical and laboratory parameters between patients with axSpA and healthy controls. Overall correlation heatmaps revealed distinct networks of association among immune cell subsets, acute-phase reactants, and metabolic markers, defined here as serum lipid profile components including TG, TC, LDL, APOB, and APOE (**Figures 2A, B**). In axSpA patients, the proportion of Tang cells was significantly negatively correlated with the disease activity index BASDAI (r = −0.548, *P* < 0.001; **Figure 2C**) but positively correlated with the proportion of CD14^+^HLA-DR^+^ cells (r = 0.537, *P* < 0.001; **Figure 2D**). Notably, no significant correlations were observed between Tang cells and ASDAS or conventional inflammatory markers, including ESR and CRP. A weak but significant negative correlation was found with apolipoprotein B (ApoB) (r = -0.291, *P* = 0.034). In contrast, among healthy controls, Tang cell levels showed significant correlations with the percentage of Treg cells, triglyceride (TG) levels, and apolipoprotein E (APOE) levels.

**Figure 2:**
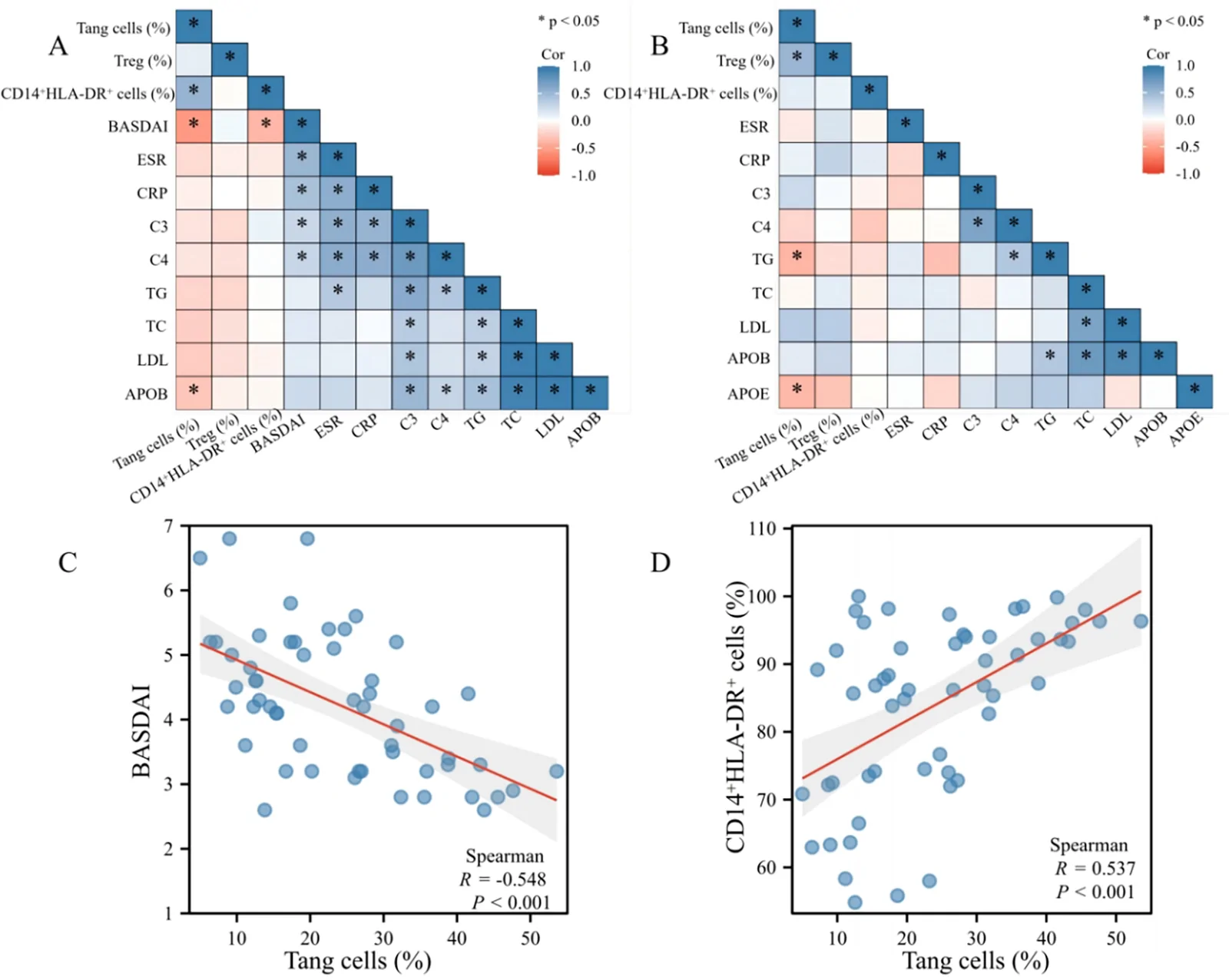
The results of the correlation analysis. **(A)** Correlation analysis among various immune markers, inflammatory markers, and lipid markers in the axSpA patient group. **(B)** Correlation matrix of the same markers in the healthy control group. The heatmap colors range from a blue gradient (representing positive correlation) to a red gradient (representing negative correlation), * indicates *p* < 0.05, denoting statistical significance. **(C)** The correlation analysis curve plot of Tang cell proportion and BASDAI. **(D)** The correlation analysis curve plot of Tang cell proportion and CD14^+^HLA-DR^+^ proportion. TG, triglycerides; TC, total cholesterol; LDL-C, low-density lipoprotein cholesterol; ApoB, Apolipoprotein B; ESR: Erythrocyte sedimentation rate; CRP, C-Reactive protein; C3, Complement component 3; C4, Complement component 4.

### Tang cell proportion as a novel independent predictor for axSpA activity

The findings from the multivariable logistic regression analysis on axSpA disease activity-associated factors are summarized in **Table 4**. In the multivariable model, only Tang cell frequency was independently and inversely associated with active disease status(adjusted OR = 0.87, 95% CI: 0.79-0.95, *P* = 0.002). No statistically significant associations were observed for sex, age, ESR, or CRP (all 95% CIs included 1; all *P* > 0.05). Receiver Operating Characteristic (ROC) curve analysis was performed for Tang cell percentage, ESR, CRP, and CD14^+^HLA-DR^+^ cells. Pairwise comparisons of AUCs using DeLong’s test indicated no significant differences in discriminatory capacity among Tang cells, ESR, and CRP (all *P* > 0.05). However, compared with CD14^+^HLA-DR^+^ cells, only Tang cell percentage demonstrated a significantly higher AUC (Z = 2.008, *P* = 0.045), whereas neither ESR (Z = 0.650, *P* = 0.516) nor CRP (Z = 0.015, *P* = 0.988) showed statistically significant differences (**Figure 3**).

**Table 4:** Multivariable logistic regression to identify predictors of disease activity in axSpA.

Variables	Multivariate regression analyses		
	β	OR (95% CI)	P
Sex (Male vs. Female)	0.64	1.90(0.24-14.77)	0.540
Age(year)	0.05	1.05(0.98-1.13)	0.134
ESR (mm/h)	0.08	1.08(0.99-1.17)	0.067
CRP (mg/dL)	-0.04	0.96(0.85-1.08)	0.494
Tang cells(%)	-0.14	0.87(0.79-0.95)	0.002

OR, Odds Ratio; CI, Confidence Interval; ESR, Erythrocyte sedimentation rate; CRP, C-Reactive protein.

**Figure 3:**
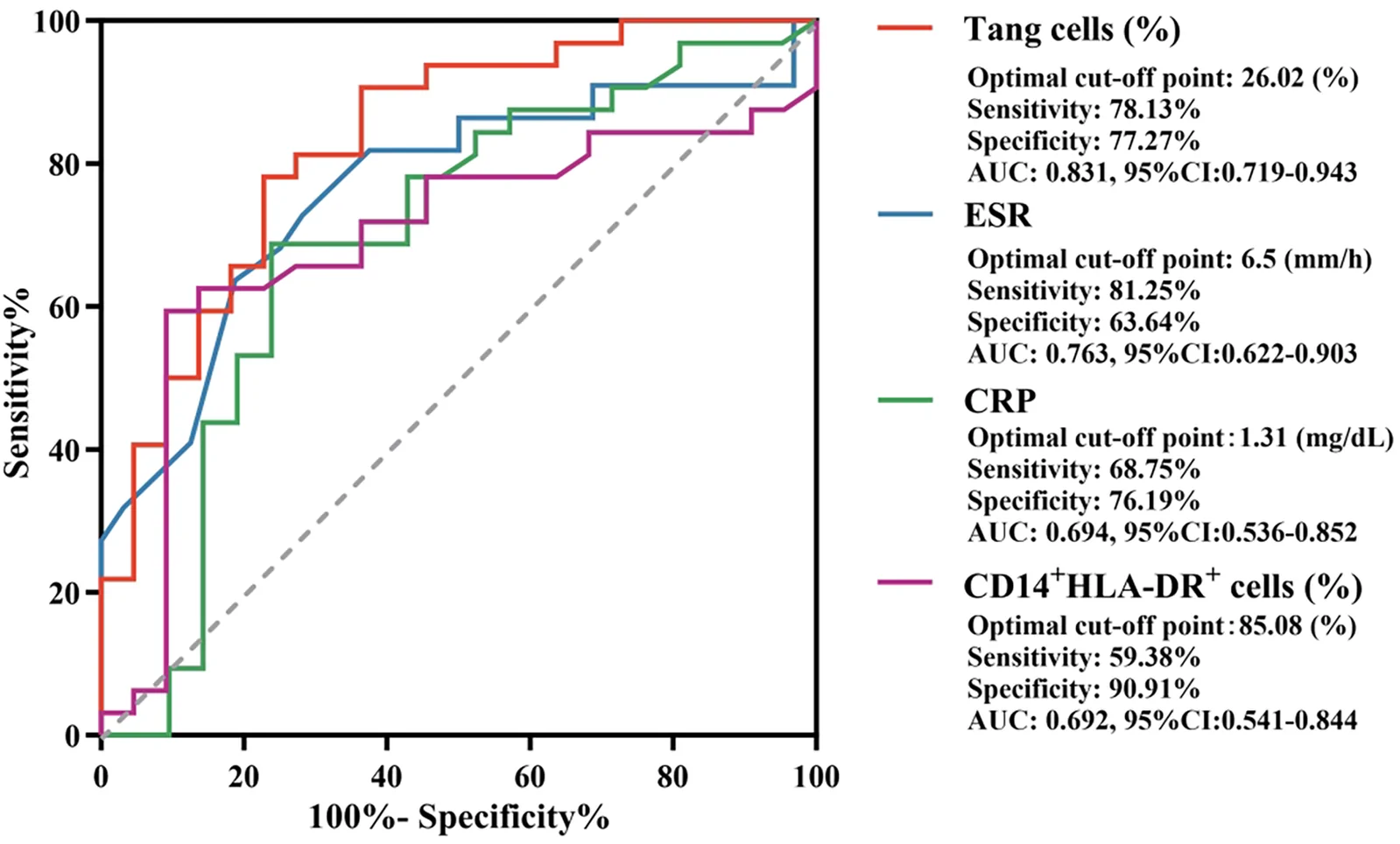
Comparison of ROC Curves for Tang Cell Percentage, ESR, CRP, CD14^+^HLA-DR^+^ cells and in Predicting Disease Activity in axial spondyloarthritis. OR, Odds Ratio; CI, Confidence Interval; ESR, Erythrocyte sedimentation rate; CRP, C-Reactive protein.

## Discussion

In recent years, a growing body of evidence has focused on the potential mechanisms underlying the increased cardiovascular risk and vascular endothelial dysfunction observed in patients with axSpA (21, 22). In this context, the present study provides novel clinical evidence linking Tang cells to axSpA disease activity. We demonstrate for the first time that the proportion of circulating Tang cells is significantly reduced in patients with active axSpA and is inversely correlated with disease activity as assessed by the BASDAI. While showing comparable discriminatory capacity to ESR and CRP in ROC analysis, circulating Tang cell frequency was independently and inversely associated with active disease status in multivariable analysis. This core finding suggests a potential immune-vascular dimension associated with axSpA disease activity, which may be reflected by alterations in circulating Tang cells and complementary to the information provided by conventional inflammatory markers.

When interpreting these findings, it is important to consider the baseline clinical and genetic characteristics of the study population. The demographic profile of our cohort is consistent with that of classical axSpA populations reported in previous studies, including a high prevalence of HLA-B27 positivity, confirming the representativeness of the enrolled patients (23). Our analysis revealed a concurrent increase in Tregs and a decrease in CD14^+^HLA-DR^+^ monocytes in axSpA patients, a pattern suggestive of an altered immune state under chronic inflammation. The elevated Tregs may reflect a compensatory immunoregulatory response aimed at limiting excessive inflammation, potentially influenced by therapy or endogenous regulatory mechanisms. However, circulating Treg levels in axSpA have been reported as increased, decreased, or unchanged across studies, largely due to differences in the selection of surface-specific Treg markers (24). As expected, patients with active axSpA exhibited significantly higher BASDAI and ASDAS scores than those with stable disease. Nevertheless, the clinical utility of these disease activity measures requires cautious interpretation. BASDAI is inherently subjective and dependent on patient-reported symptoms, while ASDAS may underestimate disease activity in a subset of patients with prominent clinical manifestations but normal CRP levels (25, 26). Against this background, the identification of objective, immune-based biomarkers capable of complementing current assessment tools is of particular clinical interest. In line with this concept, the observed differences in hematological, biochemical, and inflammatory parameters among the study groups reflect the systemic inflammatory and metabolic burden associated with axSpA.

Tang cells were originally characterized as a distinct T-cell subset co-expressing CD3, CD31, and CXCR4 (CD184), with established roles in endothelial repair and angiogenic processes through the secretion of pro-angiogenic mediators and direct interactions with endothelial cells (27). Dysregulation in both the number and function of Tang cells has been reported in various autoimmune and inflammatory diseases and has been implicated in disease activity and tissue injury (28). Despite increasing recognition of endothelial dysfunction in axSpA, the role of Tang cells in this disease has remained largely unexplored (17–19). In this study, we systematically evaluated circulating Tang cells in axSpA patients stratified by disease activity. Our results indicate that Tang cells are selectively reduced in patients with active disease, suggesting a critical impairment of vascular reparative function despite a state of generalized systemic inflammation. Previous studies investigating Tang cells in other autoimmune conditions have reported heterogeneous findings. In systemic lupus erythematosus, both increased and decreased Tang cell frequencies have been described depending on disease activity and cellular senescence status (19, 29). In systemic sclerosis, increased Tang cell numbers have been interpreted as a compensatory response to ongoing vascular injury, particularly in patients with digital ulcers (17). Similarly, reduced Tang cell levels have been associated with impaired pulmonary function in autoimmune-related interstitial lung disease (30). In the context of axSpA, our findings suggest that the marked reduction of Tang cells in active disease may indicate impaired chemotactic capacity, altered homing behavior, or functional exhaustion within an inflammatory microenvironment.These interpretations should be considered hypothesis-generating and require further mechanistic validation.

The most robust observation in the present study is the significant inverse correlation between Tang cell proportion and axSpA disease activity, as measured by BASDAI. This finding is consistent with reports in rheumatoid arthritis, where reduced Tang cell frequencies were similarly associated with higher disease activity scores (18). Interestingly, while Tang cell proportion was significantly correlated with BASDAI, it did not show a significant association with ASDAS. This apparent discrepancy likely reflects the different dimensions captured by these assessment tools. BASDAI is a patient-reported outcome that integrates multiple subjective symptoms, including fatigue, spinal and peripheral pain, and morning stiffness, which may be more sensitive to immune-mediated tissue-level inflammation and endothelial repair demands. In contrast, ASDAS relies heavily on objective inflammatory markers, particularly CRP levels, which predominantly reflect systemic, cytokine-driven inflammation. Notably, Tang cell proportion did not exhibit a significant correlation with classical systemic inflammatory markers, such as ESR or CRP, a phenomenon also reported in a previous study (29). This dissociation may reflect fundamental differences in the biological processes captured by these markers. ESR and CRP predominantly reflect generalized, cytokine-driven systemic inflammation, whereas Tang cells represent a specialized immune population involved in vascular repair and angiogenic regulation (31, 32). Consequently, Tang cell dynamics may be influenced by local tissue environments and reparative demands, rather than by acute systemic inflammatory responses alone, which may explain their selective correlation with BASDAI.

Another important finding of this study is the significant positive correlation between Tang cell proportion and the frequency of CD14^+^HLA-DR^+^ cells. HLA-DR expression on CD14^+^ monocyte-lineage cells is widely recognized as an indicator of innate immune activation and antigen-presenting capacity. Tang cells, in contrast, are considered immune-vascular phenotype markers associated with endothelial homeostasis. Across chronic inflammatory states and malignancies, circulating monocytes frequently downregulate surface HLA-DR, resulting in an expansion of CD14^+^HLA-DR^-/low^ immunosuppressive subpopulations (33, 34). Notably, recent evidence in axSpA demonstrates a similar monocyte subset imbalance, where a depletion of circulating CD14^+^CD11c^+^HLA-II^+^ monocytes occurs alongside a reciprocal expansion of an HLA-II^-^ subset that correlates with local MRI inflammatory scores, implicating these HLA-II^-^ subpopulations in active disease progression (35). Aligning with these observations, the reduction in CD14^+^HLA-DR^+^ monocytes in active axSpA in our cohort likely reflects a shift in monocyte heterogeneity toward an HLA-D^-/low^ phenotype. Together, the shift toward immunosuppressive monocyte phenotypes and the reduction in Tang cell counts reflect a parallel impairment of functional innate immunity and vascular repair capacity.

Furthermore, we observed a weak inverse correlation between circulating Tang cells and APOB levels. Tang cells reflect endothelial repair capacity and vascular homeostasis (27), whereas APOB indexes total circulating atherogenic particle burden (36). Therefore, the observed inverse correlation may reflect the coexistence of reduced vascular repair-associated cellular profiles and altered lipid metabolism under chronic inflammatory conditions. However, given the cross-sectional design of our study, this association should not be interpreted as a direct causal relationship. Notably, although APOB levels were slightly higher in patients with active disease than in those with stable disease, values across all axSpA subgroups remained within normal clinical reference ranges. These findings align with the established ‘lipid paradox’ in spondyloarthritis, wherein active systemic inflammation accelerates cardiovascular risk despite normal or paradoxically low circulating atherogenic lipid profiles, such as total and LDL cholesterol (37, 38). This suggests that vascular risk in active axSpA may relate more to inflammation-driven alterations in lipoprotein function and impaired endothelial protection than to quantitative apolipoprotein accumulation. Thus, as a potential circulating marker of endothelial repair capacity, Tang cells may provide complementary insights into early vascular injury beyond traditional lipid parameters.

A multivariable logistic regression model was constructed, adjusting for age, sex, ESR, and CRP. Notably, while ESR and CRP lost statistical significance in this comprehensive model, Tang cell frequency remained independently and inversely associated with active disease status. ROC curve analysis showed that the discriminatory efficacy of Tang cell percentage was comparable to that of ESR and CRP for identifying active axSpA. Taken together, these analytical perspectives suggest that Tang cells may serve as a potential complementary indicator for axSpA disease activity assessment, offering an insightful immunological perspective on disease stratification.

Finally, several limitations of this study should be acknowledged. The relatively small sample size and single-center design may restrict the generalizability of the findings. In addition, flow cytometry panel constraints, specifically the lack of CD16, prevented further characterization of classical, intermediate, and nonclassical monocyte subsets, as well as the evaluation of precise HLA-DR expression intensity. Furthermore, detailed characterization of disease-relevant lymphocyte subsets, such as Th17 cells and group 3 innate lymphoid cells, was not performed. Future studies incorporating more comprehensive immunophenotyping may provide deeper mechanistic insights into AS immunopathology. Moreover, the cross-sectional nature of the study precludes conclusions regarding causality or temporal relationships between Tang cell alterations and disease activity. Furthermore, treatment was not adjusted for in our multivariable analysis, which represents a potential confounding factor given that b/tsDMARDs were used in 90.9% of stable patients compared with 75.0% of active patients. Future multicenter, longitudinal studies incorporating treatment stratification are warranted to validate Tang cells as biomarkers and to clarify their potential role in monitoring disease progression and therapeutic response. Additionally, because the optimal cut-off value of 26.02% for Tang cells was derived and evaluated within the same cohort, its diagnostic performance metrics may be subject to optimism bias and require external prospective validation. Despite these limitations, the present study provides new clinical evidence linking Tang cell subset imbalance to axSpA disease activity and offers a foundation for future investigations into immune-mediated vascular dysregulation in axSpA.

## Conclusions

In conclusion, this study systematically characterizes circulating angiogenic T cells in axial spondyloarthritis for the first time in relation to disease activity and demonstrates that their proportion is significantly reduced in active disease. Tang cell frequency was independently associated with disease activity after adjustment for conventional inflammatory markers, highlighting its unique and non-redundant immunological value. Although Tang cell frequency exhibited comparable discriminatory performance to ESR and CRP in ROC analysis, multivariable modeling revealed that it remained independently and inversely associated with active disease status, suggesting a biological feature distinct from traditional systemic inflammatory markers. These findings suggest that axSpA disease activity is not solely driven by systemic inflammation but is also associated with alterations in vascular reparative immune compartments. Collectively, our results provide a novel immune-vascular perspective on axSpA pathogenesis and support Tang cells as a promising candidate biomarker that complements current activity assessment tools. Further longitudinal and multicenter investigations are required to validate their prognostic utility and to elucidate the mechanistic basis linking Tang cell dysregulation to disease progression.

## Data Availability

The raw data supporting the conclusions of this article will be made available by the authors, without undue reservation.
